# Supplementary material for: Assembly and lipid-gating of LRRC8A:D volume-regulated anion channels
Source: Nat Commun. 2025 Dec 12;17:366. doi: 10.1038/s41467-025-67052-5 (PMC12795811; doi:10.1038/s41467-025-67052-5)
Supplement: Supplementary file 1 — Supplementary Information [file 41467_2025_67052_MOESM1_ESM.pdf]

Supplementary Information

Assembly and lipid-gating of LRRC8A:D volume-regulated anion channels

Antony Lurie[1,2], Christina A. Stephens[3], David M. Kern[1,2,‡], Katharine M. Henn[4], Naomi R. Latorraca[3], & Stephen G. Brohawn[1,2,4]*

1. Department of Molecular & Cell Biology, University of California, Berkeley, CA, USA
2. California Institute for Quantitative Biology (QB3), University of California, Berkeley, CA, USA
3. Department of Biochemistry and Molecular Biophysics, Columbia University Irving Medical Center, New York, NY, USA
4. Department of Neuroscience, University of California, Berkeley, CA, USA

‡Current address: ModeX Therapeutics, Weston, MA, USA

*Correspondence to brohawn@berkeley.edu

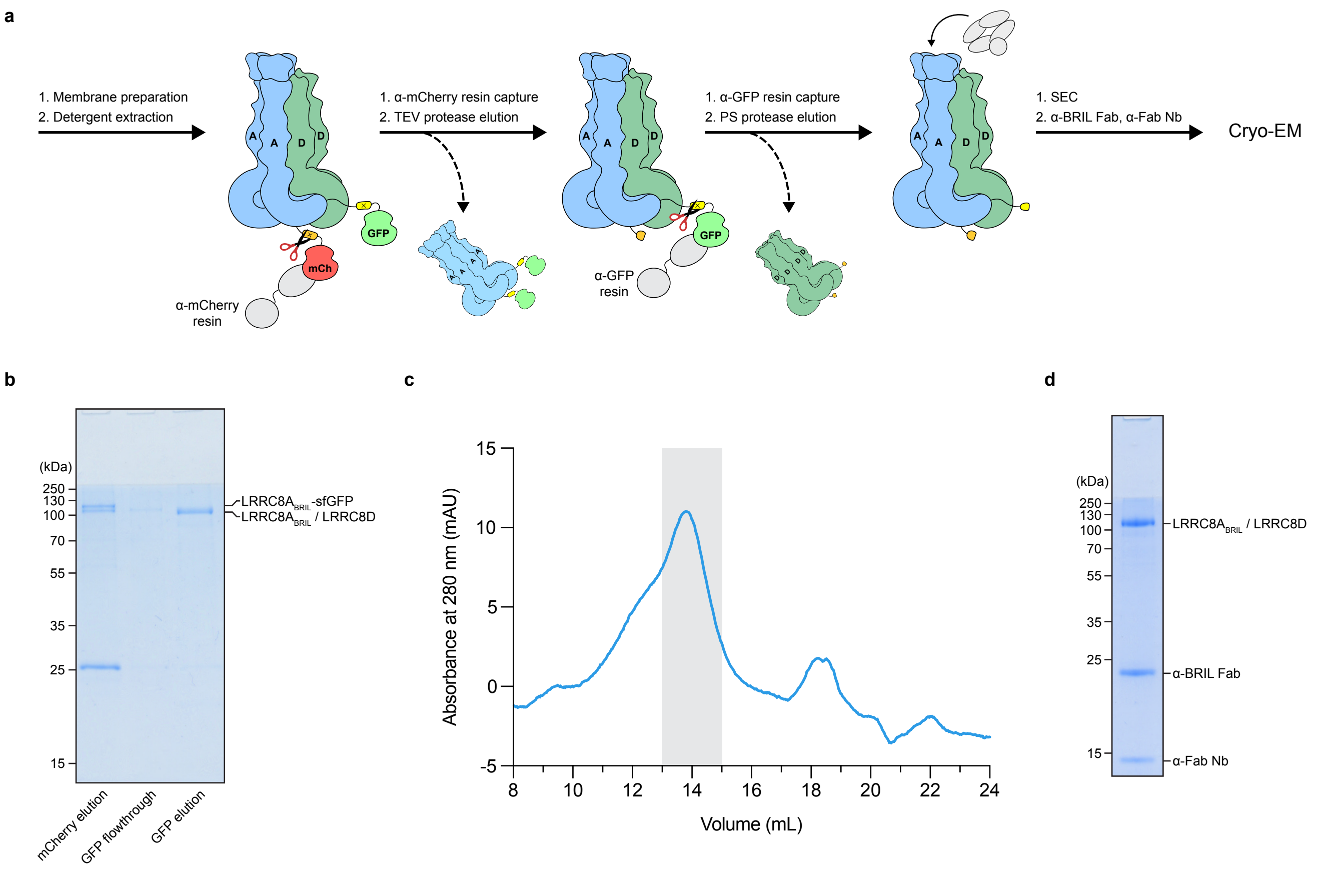

Supplementary Figure 1. Purification of LRRC8A$_{BRIL}$:D VRACs. a) Schematic of the LRRC8A$_{BRIL}$:D purification approach. **b)** Coomassie-stained SDS-PAGE of LRRC8A$_{BRIL}$:D purification samples following elution from the mCherry Nb resin (*left*), flow-through after GFP Nb resin binding (*center*), and elution from the GFP Nb resin (*right*). **c)** Size-exclusion chromatogram of the crude LRRC8A$_{BRIL}$:D sample (blue line, monitoring by absorbance at 280 nm) with pooled sample fractions highlighted in gray. **d)** Coomassie-stained SDS-PAGE of the final LRRC8A$_{BRIL}$:D sample with added α-BRIL Fab (BAG2) and α-Fab Nb. mCh, mCherry; PS, PreScission; SEC, size-exclusion chromatography.

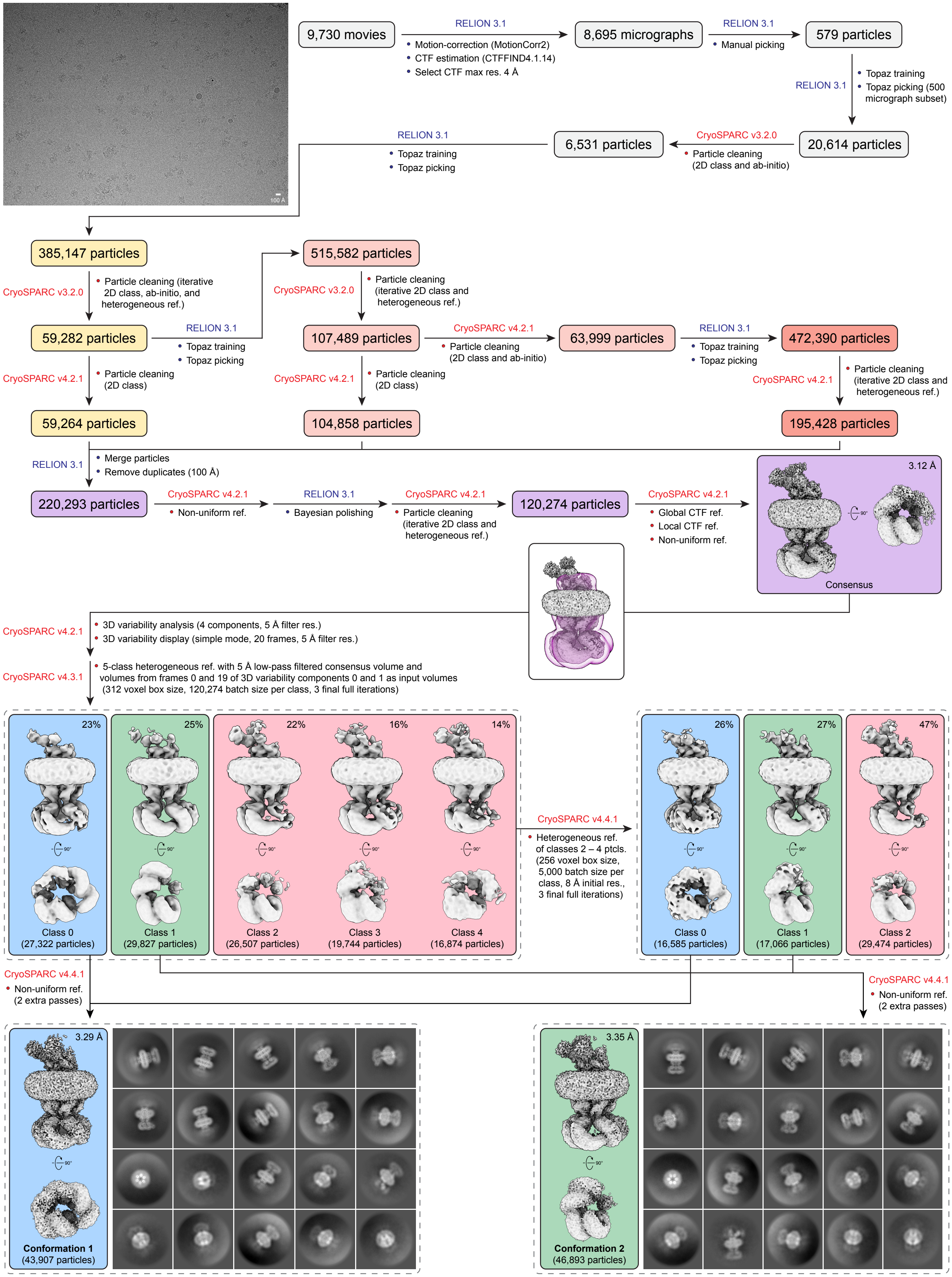

Supplementary Figure 2. Summarized workflow for cryo-EM data processing. A representative micrograph is displayed in the top left corner. Representative 2D classes from the final particle stacks are displayed on the bottom. Ref., refinement; res., resolution.

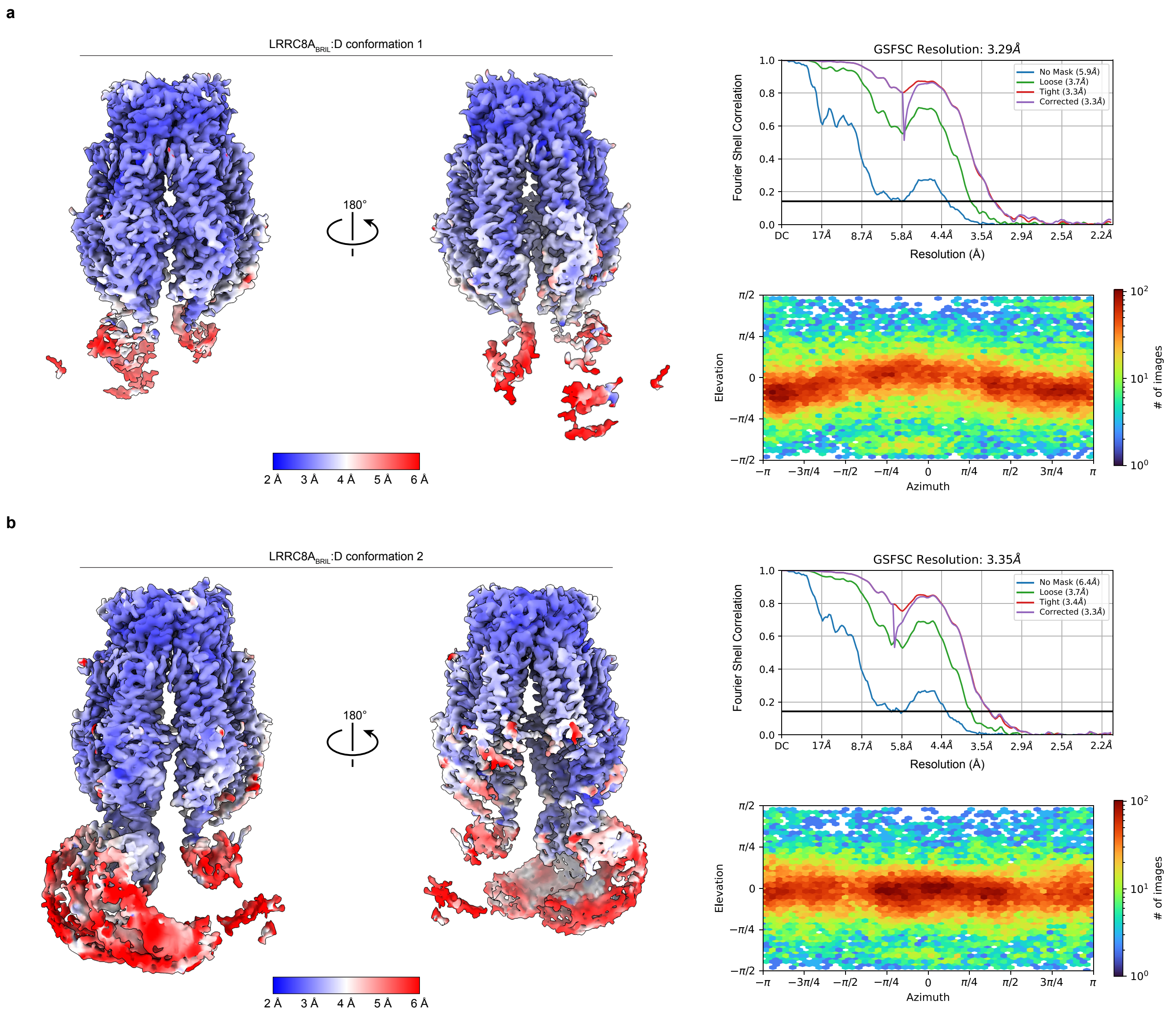

Supplementary Figure 3. Validation of cryo-EM data. Two side views of map density colored by local resolution (*left*), Fourier shell correlation (FSC) plots (*right*, *top*), and viewing direction distribution plots (*right*, *bottom*) for LRRC8A:D conformation 1 (**a**) and 2 (**b**).

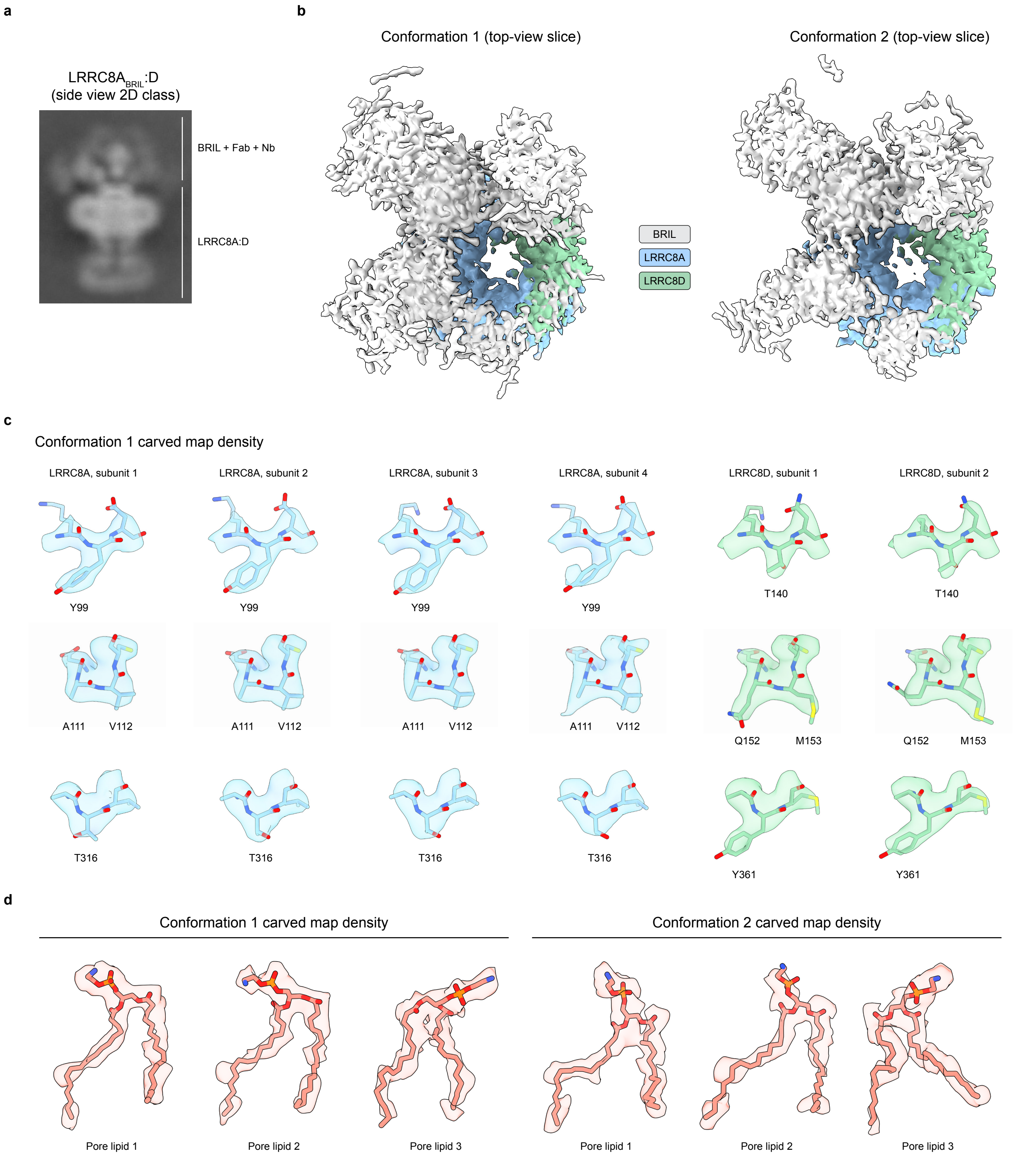

Supplementary Figure 4. Validation of subunit assignment and modeled pore lipids. a) 2D class of LRRC8A$_{BRIL}$:D exhibiting density for BRIL / α-BRIL Fab / α-Fab Nb complexes. **b)** Top-view slices of the LRRC8A:D conformation 1 (*left*) and 2 (*right*) maps focusing on the extracellular region (LRRC8A, blue; LRRC8D, green) and globular BRIL densities (gray), highlighting density for a BRIL domain positioned above each of the four LRRC8A$_{BRIL}$ subunits. **c)** Selected residues with sequence differences between LRRC8A and LRRC8D overlaid with their carved cryo-EM map densities. The residue comparisons displayed are: Y99 (LRRC8A) to T140 (LRRC8D), A111–V112 (LRRC8A) to Q152–M153 (LRRC8D), and T316 (LRRC8A) to Y361 (LRRC8D). **d)** Modeled pore lipids for LRRC8A:D conformation 1 (*left*) and 2 (*right*) overlaid with their carved cryo-EM map densities.

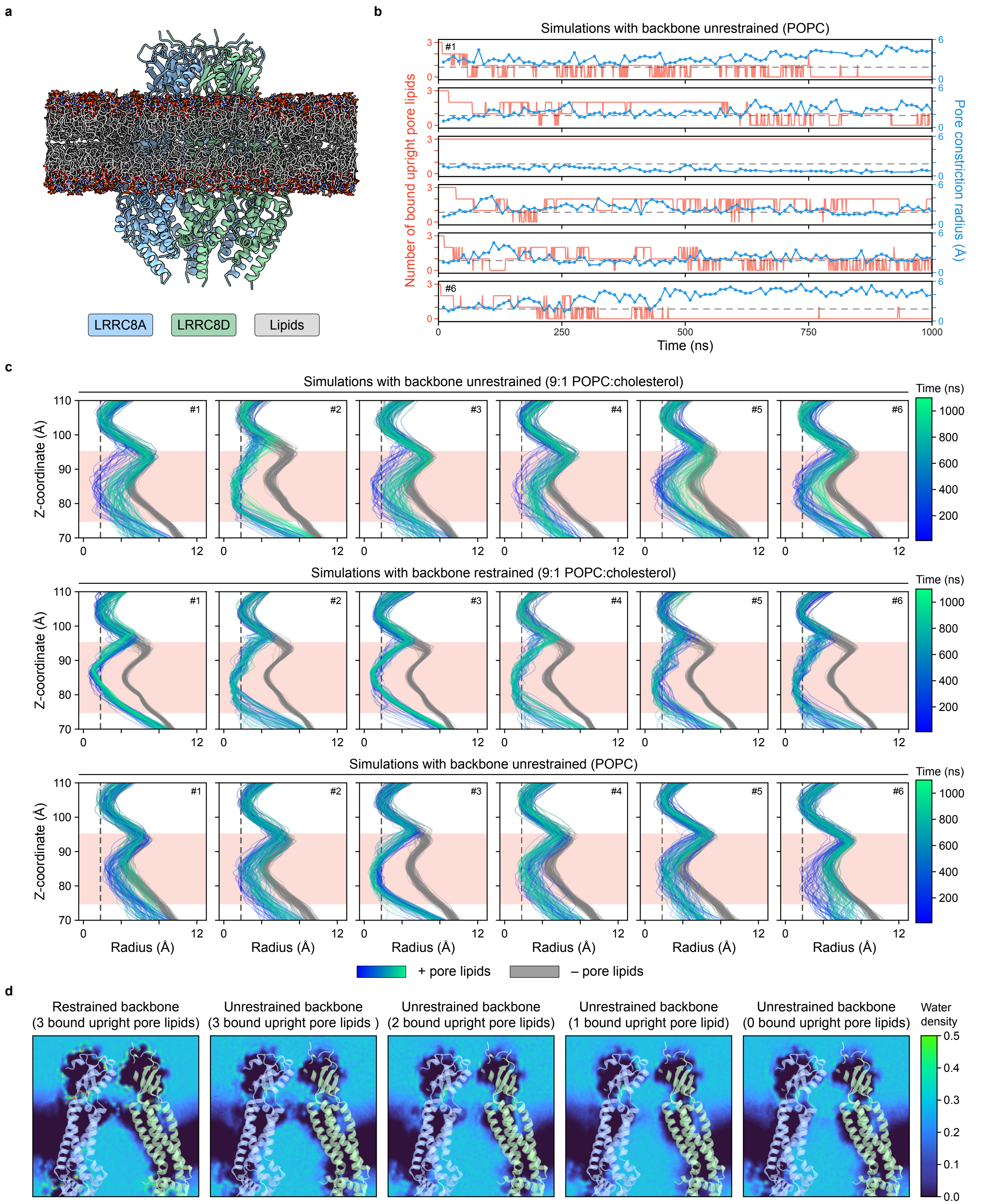

Supplementary Figure 5. Molecular dynamics simulations of pore lipids in LRRC8A:D. a) Side-view of the prepared simulation box with LRRC8A:D embedded in a lipid bilayer. LRRC8A subunits, blue; LRRC8D subunits, green; membrane lipids (POPC, cholesterol), gray. **b)** Time traces from backbone unrestrained simulations in POPC-only membranes, indicating the number of lipids that remain bound upright within the pore (salmon; simulations sampled every 1 ns) and the measured radius of the pore (Å) at the constriction site (blue; simulations analyzed every 10 ns, as described in the Methods). The radius for a bare Cl^- ion (1.8 Å) is indicated with a gray dashed line. Individual simulation replicates (#1 – 6) are plotted successively from top to bottom. **c)** Pore profiles determined across simulation frames for backbone unrestrained simulations in 9:1 POPC:cholesterol membranes (*top*), backbone restrained simulations in 9:1 POPC:cholesterol membranes (*middle*), and backbone unrestrained simulations in POPC-only membranes (*bottom*). Pore radii (Å) are plotted as a function of z-position within the pore (Å), with pore lipids included (solid lines colored from blue to green with increasing simulation time) or excluded (gray lines) from the pore radius calculation. Individual simulation replicates (#1 – 6) are plotted successively from left to right. **d)** Water occupancy calculated across unrestrained and restrained simulations separately and clustered by numbers of bound upright lipids in the pore, reported as the average number of water molecules within a grid of 1 $Å^3$ and analyzed every 1 ns.

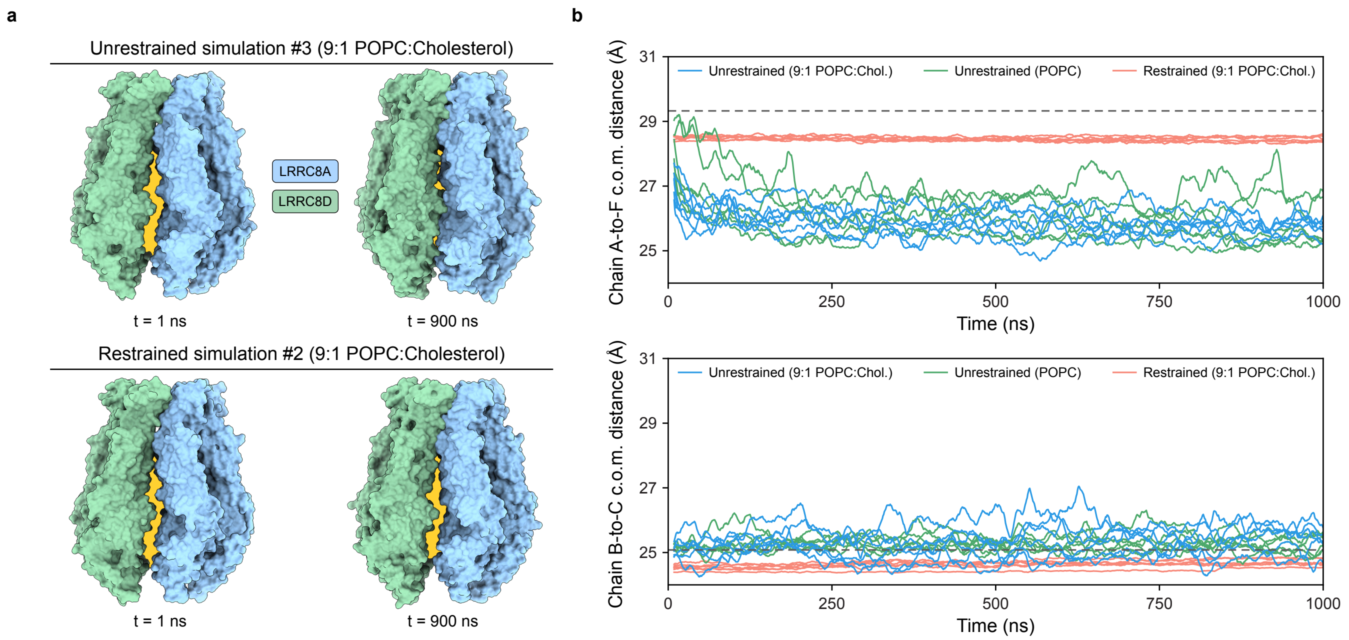

Supplementary Figure 6. Intersubunit distances in simulations of LRRC8A:D. a) Side-views of LRRC8A:D immediately after the end of equilibration (1 ns) and near the end of the total simulation (900 ns) for an unrestrained (*top*) and restrained (*bottom*) simulation in 9:1 POPC:cholesterol membranes. The intersubunit gap between LRRC8A (blue) and LRRC8D (green) subunits is highlighted in yellow. **b)** Calculated distances between the centers of mass for the transmembrane regions of LRRC8D (chain F) and LRRC8A (chain A) (*top*) or between two LRRC8A subunits (chains A and B) (*bottom*). Data from each simulation are plotted as rolling averages computed over a moving 10-ns window with simulation data sampled every 1 ns. Distances are plotted for unrestrained simulations in 9:1 POPC:cholesterol membranes in blue, unrestrained simulations in POPC-only membranes in green, and restrained simulation in 9:1 POPC:cholesterol membranes in salmon. The LRRC8D–LRRC8A distance in the starting cryo-EM structure (Conformation 1) is shown as a gray dashed line.

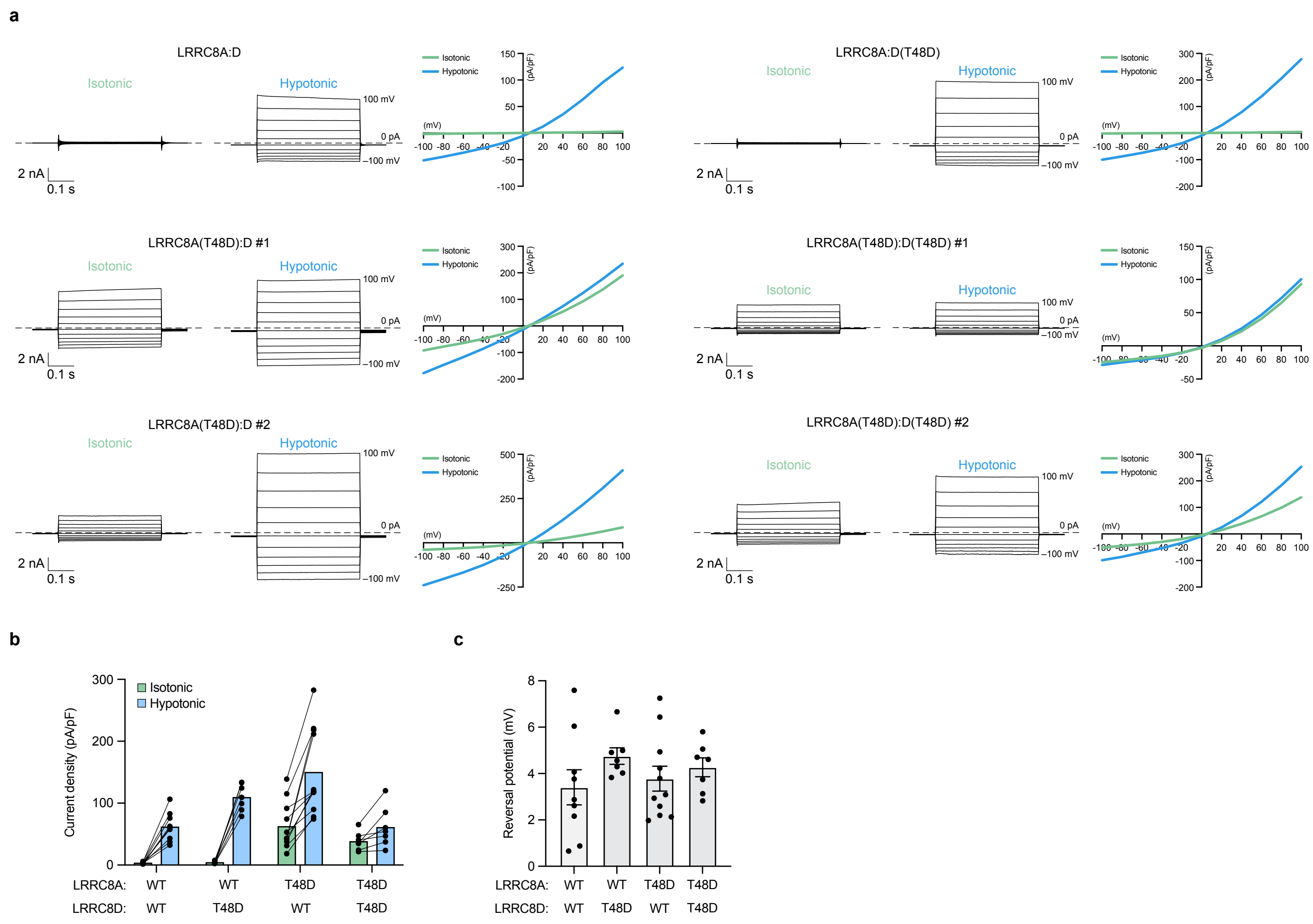

Supplementary Figure 7. Electrophysiology of LRRC8A:D lipid gate mutants. a) Whole-cell voltage-clamp recordings from *LRRC8A-E*$^{-/-}$ HeLa cells expressing wild-type (WT) LRRC8A:D, LRRC8A:D(T48D), LRRCA(T48D):D, or LRRC8A(T48D):D(T48D) mutant channels. For each, representative current traces from isotonic (*left*) and hypotonic (*center*) solutions are displayed alongside corresponding plots of the current-voltage relationships (*right*; isotonic, green; hypotonic, blue). Due to variability in the observed fold activation, two representative examples are shown for LRRCA(T48D):D and LRRC8A(T48D):D(T48D). For current traces, 0 pA/pF is marked with a dotted line. **b)** Current densities for WT LRRC8A:D (n = 9), LRRC8A:D(T48D) (n = 7), LRRC8A(T48D):D (n = 11), and LRRC8A(T48D):D(T48D) (n = 7) channels in isotonic (green) and hypotonic (blue) solutions. Data are displayed as the mean plotted alongside paired individual data points for each cell. **c)** Reversal potentials following hypotonic swelling. Data are displayed as the mean ± s.e.m. plotted alongside individual data points for LRRC8A:D (n = 9), LRRC8A:D(T48D) (n = 7), LRRC8A(T48D):D (n = 11), and LRRC8A(T48D):D(T48D) (n = 7).

Supplementary Table 1. Cryo-EM data collection, refinement, and validation statistics

	LRRC8A:D Conformation 1<br><br>EMDB-47282<br>PDB 9DX7<br>EMPIAR-12510	LRRC8A:D Conformation 2<br><br>EMDB-47283<br>PDB 9DXA<br>EMPIAR-12510
**Data collection and processing**		
Magnification	81,000	81,000
Voltage (kV)	300	300
Electron exposure ($e^{–}/Å^2$)	50	50
Defocus range (μm)	-0.6 to -1.6	-0.6 to -1.6
Pixel size (Å)	1.048	1.048
Initial movies (no.)	9730	9730
Final movies (no.)	8695	8695
Symmetry imposed	*C*1	*C*1
Initial particle images (no.)	220,293	220,293
Final particle images (no.)	43,907	46,893
Map resolution (Å)	3.29	3.35
FSC threshold	0.143	0.143
Map resolution range (Å)	2.3 – 44.5	2.3 – 43.7
**Refinement**		
Initial model used (PDB code)	8DS3	9DX7
Model resolution (Å)	3.2	3.3
FSC threshold	0.143	0.143
Model resolution range (Å)	2.3 – 6.4	2.3 – 7.9
Map sharpening *B* factor ($Å^2$)	–42.9	–43.2
Model composition		
Non-hydrogen atoms	16,238	16,291
Protein residues	1,873	1,880
Ligands	18	18
B factors ($Å^2$)		
Protein	99.27	107.04
Ligand	92.74	84.30
R.m.s. deviations		
Bond lengths (Å)	0.002	0.002
Bond angles (°)	0.424	0.436
Validation		
MolProbity score	1.22	1.33
Clashscore	4.41	4.61
Poor rotamers (%)	0.17	0.23
Ramachandran plot		
Favored (%)	97.99	97.56
Allowed (%)	2.01	2.44
Disallowed (%)	0.00	0.00

Supplementary Table 2. Details of molecular dynamics simulations.

System preparation method	Replica number	Box dimensions	Number of atoms	Total number of water molecules	Salt concentration (NaCl)	Lipid Composition
Prime + Dowser + PACKMOL-Memgen + AmberTools 24	1 – 6	145 Å × 146 Å × 164 Å	391,424	72,766	150 mM	9:1 (n=728:53) POPC/CHOL
CHARMMGUI	1	140 Å × 140 Å × 166 Å	377,384	72,012	150 mM	POPC (n=425)
	2	140 Å × 140 Å × 166 Å	378,784	72,372	150 mM	POPC (n=424)
	3	140 Å × 140 Å × 166 Å	378,690	72,320	150 mM	POPC (n=425)
	4	140 Å × 140 Å × 166 Å	378,958	72,320	150 mM	POPC (n=427)
	5	140 Å × 140 Å × 166 Å	378,636	72,340	150 mM	POPC (n=424)
	6	140 Å × 140 Å × 166 Å	379,368	72,389	150 mM	POPC (n=428)

Supplementary Table 3. Molecular dynamics simulations checklist.

**Reliability and reproducibility checklist for molecular dynamics simulations**<br>***All boxes must be marked YES by acceptance unless "Response not needed if No".**	**Yes**	**No**	**Response**<br>**(Please state where this information can be found in the text)**
**1. Convergence of simulations and analysis**			
1a. Is an evaluation presented in the text to show that the property being measured has equilibrated in the simulations<br>(*e.g.* time-course analysis)?	☒	☐	We show time-courses for each simulation included in our analysis in Figure 4A and Supplementary Figures 5B and 6B.
1b. Then, is it described in the text how simulations are split into equilibration and production runs and how much data were analyzed from production runs?	☒	☐	In our Methods section 'Molecular dynamics simulations', we indicate the length of each equilibration step and note that our analysis includes all portions of production runs.
1c. Are there at least 3 simulations per simulation condition with statistical analysis?	☒	☐	In our Methods section 'Molecular dynamics simulations', we describe each simulation condition, and in Figure 4A and Supplementary Figure 5B we show time traces for the 6 replicas per condition.
1d. Is evidence provided in the text that the simulation results presented are independent of initial configuration?	☒	☐	In the time-courses for each simulation without protein restraints in Figure 4A and Supplementary Figure 5B, two simulations result in steady bound-lipid positions despite different initial water and bulk lipid positions.
**2. Connection to experiments**			
2a. Are calculations provided that can connect to experiments (*e.g.* loss or gain in function from mutagenesis, binding assays, NMR chemical shifts, J-couplings, SAXS curves, interaction distances or FRET distances, structure factors, diffusion coefficients, bulk modulus and other mechanical properties, *etc.*)?	☒	☐	Measurement of the pore radius in our simulations relates to the changes in conductance seen experimentally.
**3. Method choice**			
3a. Do simulations contain membranes, membrane proteins, intrinsically disordered proteins, glycans, nucleic acids, polymers, or cryptic ligand binding?	☒	☐	All our simulations are of the LRRC8A:D membrane protein in a pure POPC or POPC/cholesterol membrane bilayer as described in the Methods.
3b. Is it described in the text whether the accuracy of the chosen model(s) is sufficient to address the question(s) under investigation (e.g. all-atom vs. coarse-grained models, fixed charge vs. polarizable force fields, implicit vs. explicit solvent or membrane, force field and water model, etc.)?	☒	☐	We describe two simulation preparation methods we used to diversify our simulation initial conditions as described in the Methods. In the Results section describing our simulation setup we motivate the use of fully atomistic MD.

3c. Is the timescale of the event(s) under investigation beyond the brute-force MD simulation timescale in this study that enhanced sampling methods are needed?		☐	☒	
	If **YES**, are the parameters and convergence criteria for the enhanced sampling method clearly stated?	☐	☐	
	If **NO,** is the evidence provided in the text?	☒	☐	Our goal was to test the stability of lipids bound within the protein pore and we observed many dissociation events within the timescale of our simulations (see Figure 4A and Supplementary Figure 5B).
**4. Code and reproducibility**				
4a. Is a table provided describing the system setup that includes simulation box dimensions, total number of atoms, total number of water molecules, salt concentration, lipid composition (number of molecules and type)?		☒	☐	We provide a detailed description of these simulated system parameters in our Methods and Supplementary Table 2.
4b. Is it described in the text what simulation and analysis software and which versions are used?		☒	☐	In the last paragraph of our Methods section ‘Molecular dynamics simulations’’.
4c. Are other parameters for the system setup described in the text, such as protonation state, type of structural restraints if applied, nonbonded cutoff, thermostat and barostat, etc.?		☒	☐	In the first paragraph of our Methods section ‘Molecular dynamics simulations’’.
4d. Are initial coordinate and simulation input files and a coordinate file of the final output provided as supplementary files or in a public repository?		☒	☐	In a Zenodo public repository: 10.5281/zenodo.16921648
4e. Is there custom code or custom force field parameters?		☐	☒	Response not needed if **No**
	If **YES**, are they provided as supplementary files or in a public repository?	☐	☐	
