# Supplementary material for: Assembly and lipid-gating of LRRC8A:D volume-regulated anion channels
Source: Nat Commun. 2025 Dec 12;17:366. doi: 10.1038/s41467-025-67052-5 (PMC12795811; doi:10.1038/s41467-025-67052-5)
Supplement: Supplementary file 2 — Description of Additional Supplementary Files [file 41467_2025_67052_MOESM2_ESM.pdf]

Description of Additional Supplementary Files

File Name: Supplementary Movie 1
Description: Side-view of the LRRC8A:D (white) pore highlighting pore lipids (salmon) and corresponding pore profiles (colored from purple (1 Å) to yellow (5 Å) by pore radius) for an unrestrained simulation in a 9:1 POPC:cholesterol membrane (#2) where pore lipids remained close to their starting positions.

File Name: Supplementary Movie 2
Description: Side-view of the LRRC8A:D (white) pore highlighting pore lipids (salmon) and corresponding pore profiles (colored from purple (1 Å) to yellow (5 Å) by pore radius) for an unrestrained simulation in a 9:1 POPC:cholesterol membrane (#3) where pore lipids dissociated from their starting positions.

File Name: Supplementary Movie 3
Description: Side-view of the LRRC8A:D (white) pore highlighting pore lipids (salmon) and corresponding pore profiles (colored from purple (1 Å) to yellow (5 Å) by pore radius) for a restrained simulation in a 9:1 POPC:cholesterol membrane (#2).
