# Supplementary material for: Assembly and lipid-gating of LRRC8A:D volume-regulated anion channels
Source: Nat Commun. 2025 Dec 12;17:366. doi: 10.1038/s41467-025-67052-5 (PMC12795811; doi:10.1038/s41467-025-67052-5)
Supplement: Supplementary file 6 — Reporting Summary [file 41467_2025_67052_MOESM6_ESM.pdf]

nature portfolio

Corresponding author(s): Stephen G. Brohawn

Last updated by author(s): 10/29/2025

Reporting Summary

Nature Portfolio wishes to improve the reproducibility of the work that we publish. This form provides structure for consistency and transparency in reporting. For further information on Nature Portfolio policies, see our Editorial Policies and the Editorial Policy Checklist.

Statistics

For all statistical analyses, confirm that the following items are present in the figure legend, table legend, main text, or Methods section.

n/a	Confirmed	
☐	☒	The exact sample size (*n*) for each experimental group/condition, given as a discrete number and unit of measurement
☐	☒	A statement on whether measurements were taken from distinct samples or whether the same sample was measured repeatedly
☐	☒	The statistical test(s) used AND whether they are one- or two-sided<br>*Only common tests should be described solely by name; describe more complex techniques in the Methods section.*
☒	☐	A description of all covariates tested
☐	☒	A description of any assumptions or corrections, such as tests of normality and adjustment for multiple comparisons
☐	☒	A full description of the statistical parameters including central tendency (e.g. means) or other basic estimates (e.g. regression coefficient) AND variation (e.g. standard deviation) or associated estimates of uncertainty (e.g. confidence intervals)
☐	☒	For null hypothesis testing, the test statistic (e.g. *F*, *t*, *r*) with confidence intervals, effect sizes, degrees of freedom and *P* value noted<br>*Give P values as exact values whenever suitable.*
☒	☐	For Bayesian analysis, information on the choice of priors and Markov chain Monte Carlo settings
☒	☐	For hierarchical and complex designs, identification of the appropriate level for tests and full reporting of outcomes
☒	☐	Estimates of effect sizes (e.g. Cohen's *d*, Pearson's *r*), indicating how they were calculated

Our web collection on statistics for biologists contains articles on many of the points above.

Software and code

Policy information about availability of computer code

Data collection	pClamp10.7, Chromlab 6.1.29, SerialEM 4.2, Schrödinger Suite (2025-1), AMBER24, AmberTools2025 (includes PACKMOL-Memgen), CHARMM-GUI (https://www.charmm-gui.org/)
Data analysis	RELION3.1, MotionCor2, CTFFIND 4.1.14, Topaz 0.2.5, cryoSPARC3-4, UCSF PyEM 0.5, Phenix 1.21, MolProbity 4.5, Coot 0.9, ChimeraX 1.9, Hole v2.2.005 implemented in Coot 0.9, AlphaFold 3, Clampfit 10.7, Prism 10.4.2, Microsoft Excel 16, Illustrator 28.1, Visual Molecular Dynamics (VMD 1.9), CAVER 3.01. Analysis scripts to run CAVER 3 on simulation data are located at: https://github.com/Latorraca-Lab/CAVER_PROTEIN_TUNNEL_PROCESSING

For manuscripts utilizing custom algorithms or software that are central to the research but not yet described in published literature, software must be made available to editors and reviewers. We strongly encourage code deposition in a community repository (e.g. GitHub). See the Nature Portfolio guidelines for submitting code & software for further information.

Data

Policy information about availability of data

All manuscripts must include a data availability statement. This statement should provide the following information, where applicable:

- Accession codes, unique identifiers, or web links for publicly available datasets
- A description of any restrictions on data availability
- For clinical datasets or third party data, please ensure that the statement adheres to our policy

Atomic coordinates are deposited in the Protein Data Bank (PDB) under accession codes 9DX7 [https://doi.org/10.2210/pdb9DX7/pdb] (LRRC8ABRIL:D conformation

1) and 9DXA [https://doi.org/10.2210/pdb9DXA/pdb] (LRRC8ABRIL:D conformation 2). Cryo-EM maps are deposited in the Electron Microscopy Data Bank (EMDB) under accession codes EMD-47282 [https://www.ebi.ac.uk/pdbe/entry/emdb/EMD-47282] (LRRC8ABRIL:D conformation 1) and EMD-47283 [https://www.ebi.ac.uk/pdbe/entry/emdb/EMD-47283] (LRRC8ABRIL:D conformation 2). Original micrograph movies and particle stacks are deposited in the Electron Microscopy Public Image Archive (EMPIAR) under accession code EMPIAR-12510 [https://doi.org/10.6019/EMPIAR-12510]. Simulation trajectories and viewing scripts are deposited in Zenodo [https://doi.org/10.5281/zenodo.16921648]. The source data for Figures 3 – 5 and Supplementary Figures 1, 3, 5 – 7 are provided as a Source Data file. Previously published models used in this manuscript were accessed from the PDB under accession codes 6G8Z [https://doi.org/10.2210/pdb6G8Z/pdb] (homomeric LRRC8A); 8DS3 [https://doi.org/10.2210/pdb8DS3/pdb] (LRRC8A:C conformation 1); and 6M04 [https://doi.org/10.2210/pdb6M04/pdb] (homomeric LRRC8D).

Research involving human participants, their data, or biological material

Policy information about studies with human participants or human data. See also policy information about sex, gender (identity/presentation), and sexual orientation and race, ethnicity and racism.

Reporting on sex and gender	n/a
Reporting on race, ethnicity, or other socially relevant groupings	n/a
Population characteristics	n/a
Recruitment	n/a
Ethics oversight	n/a

Note that full information on the approval of the study protocol must also be provided in the manuscript.

Field-specific reporting

Please select the one below that is the best fit for your research. If you are not sure, read the appropriate sections before making your selection.

☒ Life sciences ☐ Behavioural & social sciences ☐ Ecological, evolutionary & environmental sciences

For a reference copy of the document with all sections, see nature.com/documents/nr-reporting-summary-flat.pdf

Life sciences study design

All studies must disclose on these points even when the disclosure is negative.

Sample size	No statistical tests were used to predetermine sample sizes. For electrophysiology experiments a minimum of 5 independent samples was collected (standard for the field). For molecular dynamics simulations, we performed six independent replicates per simulation condition, which allowed us to observe a variety of lipid-bound and lipid-dissociated states.
Data exclusions	For cryo-EM studies, micrographs with a CTFFIND reported resolution estimate greater than 4 Å were excluded from analysis as described in the methods and processing figure. For electrophysiology experiments, recordings from cells that leaked, broke prematurely, or did not give currents following hypotonic swelling, were excluded.
Replication	Electrophysiology experiments were preformed in multiple cells from multiple biological replicates. Six replicates for each MD simulation condition were performed.
Randomization	n/a. No predetermined control and sample groups were used.
Blinding	n/a. No populations were preassigned to groups so blinding was not relevant.

Reporting for specific materials, systems and methods

We require information from authors about some types of materials, experimental systems and methods used in many studies. Here, indicate whether each material, system or method listed is relevant to your study. If you are not sure if a list item applies to your research, read the appropriate section before selecting a response.

Materials & experimental systems

n/a	Involved in the study
☒	☐ Antibodies
☐	☒ Eukaryotic cell lines
☒	☐ Palaeontology and archaeology
☒	☐ Animals and other organisms
☒	☐ Clinical data
☒	☐ Dual use research of concern
☒	☐ Plants

Methods

n/a	Involved in the study
☒	☐ ChIP-seq
☒	☐ Flow cytometry
☒	☐ MRI-based neuroimaging

Eukaryotic cell lines

Policy information about cell lines and Sex and Gender in Research

Cell line source(s)	HeLa LRRC8A-E-/- cells (Kefauver, J. M. et al. Structure of the human volume regulated anion channel. eLife 7, e38461 (2018), Sf9 (Expression Systems, 94-001F).
Authentication	We did not further authenticate the cell lines.
Mycoplasma contamination	We did not test Sf9 cells for mycoplasma. HeLa LRRC8A-E-/- cells were periodically tested and verified to be negative for mycoplasma contamination.
Commonly misidentified lines (See ICLAC register)	No commonly misidentified lines were used in our study.

Plants

Seed stocks	*Report on the source of all seed stocks or other plant material used. If applicable, state the seed stock centre and catalogue number. If plant specimens were collected from the field, describe the collection location, date and sampling procedures.*
Novel plant genotypes	*Describe the methods by which all novel plant genotypes were produced. This includes those generated by transgenic approaches, gene editing, chemical/radiation-based mutagenesis and hybridization. For transgenic lines, describe the transformation method, the number of independent lines analyzed and the generation upon which experiments were performed. For gene-edited lines, describe the editor used, the endogenous sequence targeted for editing, the targeting guide RNA sequence (if applicable) and how the editor was applied.*
Authentication	*Describe any authentication procedures for each seed stock used or novel genotype generated. Describe any experiments used to assess the effect of a mutation and, where applicable, how potential secondary effects (e.g. second site T-DNA insertions, mosiacism, off-target gene editing) were examined.*
